# Is Divorce More Painful When Couples Have Children? Evidence From Long-Term Panel Data on Multiple Domains of Well-being

**DOI:** 10.1007/s13524-016-0518-2

**Published:** 2016-11-04

**Authors:** Thomas Leopold, Matthijs Kalmijn

**Affiliations:** 1 Department of Sociology, University of Amsterdam, Nieuwe Achtergracht 166, 1018WV, Amsterdam, Netherlands; 2 Netherlands Interdisciplinary Demographic Institute (NIDI), The Hague, Netherlands

**Keywords:** Divorce, Well-being, Children, Panel data, Random-effects models

## Abstract

Theoretical models of the divorce process suggest that marital breakup is more painful in the presence of children, yet little is known about the role of children as a moderator of divorce effects on adult well-being. The present study addresses this gap of research based on long-term panel data from Germany (SOEP). Following individuals over several years before and after divorce, we investigated whether the impact of divorce on multiple measures of well-being varied by the presence and age of children before marital breakup. Three central findings emerged from the analysis. First, declines in well-being were sharper in the presence of children, and these moderator effects were larger if children were younger. Second, domain-specific measures of well-being revealed gender differences in the moderating role of children. Mothers sustained deeper drops in economic well-being than did fathers; the reverse was true for family well-being. Third, most of these disproportionate declines in the well-being of divorced parents did not persist in the long term given that higher rates of adaptation leveled out the gaps compared with childless divorcees.

## Introduction

Divorce is associated with declines in well-being and health (Hank and Wagner 2013; Kamp Dush 2013; Simon 2002, 2014; Waite 1995; Williams 2003). Such declines have been observed for several indicators, including depressive symptoms, psychological distress, and life satisfaction. Recent research has shifted the attention from estimating average effects of divorce to exploring individual heterogeneity in these effects: some breakups are especially painful, others are less so, and some might even bring relief from a stressful or unhappy situation.

Following this idea, researchers have studied moderators such as gender (Simon 2002), age (Williams and Umberson 2004), marital quality (Kalmijn and Monden 2006; Williams 2003), family background (Mandemakers et al. 2010), cohabitation versus marriage (Musick and Bumpass 2012), poverty (Liu and Chen 2006), and marriage cohort (Liu and Umberson 2008). None of these moderators have yielded consistent effects despite broad consensus that the consequences of divorce vary among the individuals involved (Amato and Anthony 2014).

Of all potential moderator variables, one of the most intriguing is whether couples have children before divorce. Although having children at home is generally believed to aggravate the effects of divorce on the well-being of former partners, only two studies have considered this moderator.^1^ A register-based study of Norwegian panel data from the early 1990s found that the detrimental effect of divorce on sickness absence (as a measure of health) was stronger when couples had children before divorce than when couples were childless (Blekesaune and Barrett 2005). An American analysis of two-wave panel data collected in 1987 and 1992 found that the increase in depression after divorce was three times stronger when couples had preschool-aged children before divorce (Williams and Dunne-Bryant 2006).

The moderating role of children is relevant for a number of reasons. First, declines in parental well-being after divorce may affect child outcomes. Many studies have shown that parental depression impacts the emotional health and psychological functioning of children (Amato and Anthony 2014; Kiernan and Huerta 2008). If the well-being of divorced parents declines more strongly and recovers less swiftly, this status shift can impose a double burden on children. Moreover, the effects of divorce and parental well-being can interact: for example, parents may be less able to provide a secure post-divorce living arrangement for their children when they have to deal with their own emotional problems.

Second, how the presence of children moderates divorce effects is relevant from a theoretical perspective. If adverse effects of divorce on the well-being of men and women are strong for parents but absent or inconsequential for childless couples, this difference has important implications for how we interpret the link between marriage and health. Traditionally, the effects of divorce on depression and other aspects of mental health have been regarded as evidence that marriage benefits health (Waite and Gallagher 2000). If such effects are limited to couples with children, however, they point to the importance of loss and crisis rather than to the role of health protection (Johnson and Wu 2002; Pearlin 2009).

In the present study, we use German panel data to examine whether and to what extent the presence of children moderates the impact of divorce on the well-being of men and women. Like the two studies before ours, we also assess the importance of child age for moderator effects, and we test how men and women differ in this respect. Apart from adding a new societal context and more recent data, we bring two new elements to this field of study. First, we strengthen the dynamic nature of the analysis. In contrast to previous research on this topic, our data include multiwave (rather than two-wave; Williams and Dunne-Bryant 2006) panel measurements and direct (rather than indirect; Blekesaune and Barrett 2005) measures of well-being. Our study draws on 29 annual waves of data from the German Socio-Economic Panel Study (SOEP), spanning the period from 1984 until 2012. These data allow us to trace changes in well-being throughout the divorce process. Important analytical benefits include a longer view of well-being before divorce and a better view of initial declines after divorce and subsequent adaptation, enabling us to determine whether—and at which point in time—people recover to predivorce levels of well-being.

Second, we contribute to the literature by looking at multiple domains of well-being. Specifically, we examine not only general life satisfaction but also satisfaction with income and satisfaction with family life. By disentangling these domains of well-being, we gain insights into the mechanisms behind the possible moderating role of children. In relation to this, we address gender differences in the effects of divorce on well-being. Men and women respond in different ways to divorce, with women experiencing more internalizing problems and men experiencing more externalizing problems (Simon 2002, 2014). We argue that men and women may also respond to divorce for different reasons. Specifically, we expect that for women, children may aggravate adverse effects of divorce via economic pathways, whereas the effect of children occurs via social pathways among men. Such a finding would suggest stronger moderator effects of children on the economic well-being of women and stronger moderator effects on the family well-being of men.

## Background and Hypotheses

Many studies have shown that the experience of a divorce negatively affects well-being and health. Studies have shifted their focus from estimating average effects to exploring heterogeneity in these effects. In Table 1, we give an overview of these studies. Gender is the most frequently studied moderator. Simon (2002) found that men are more likely to respond to divorce by exhibiting externalizing behavior (such as alcohol use), whereas women more often experience internalizing problems (such as depression). In terms of general life satisfaction, most studies have found no major gender differences, although a German study reported that men suffer more in the first years following separation (Andreß and Bröckel 2007).

**Table 1:** Overview of longitudinal studies on moderator effects of divorce on well-being since 2000, sorted by year of publication

Authors	Main Effect^a^	Outcome Studied	Moderators Studied	Moderator Effect^b^	Country	Data
Kim and McKenry (2002)	––	Depression	Gender	n.s.	USA	NSFH
Simon (2002)	––	Depression	Gender	Stronger for women	USA	NSFH
Johnson and Wu (2002)	––	Psychological distress	Marital quality	Stronger for low-quality marriages	USA	
Williams (2003)	––	Depression, life satisfaction	Gender Marital quality	n.s. Stronger for high-quality marriages	USA	ACLS
Williams and Umberson (2004)	––	Self-rated health	Age	Stronger for older persons (men) n.s. (women)	USA	ACLS
Blekesaune and Barrett (2005)	–– (women) 0 (men)	Sickness absence	Age Children	Stronger for older persons (women) Stronger for younger persons (men) Stronger for couples with children	Norway	Registeårs
Strohschein et al. (2005)	––	Psychological distress	Gender	n.s.	USA	NPHS
Liu and Chen (2006)	––	Depression	Poverty	n.s.	USA	NLSY
Kalmijn and Monden (2006)	––	Depression	Marital quality	n.s.	USA	NSFH
Williams and Dunne-Bryant (2006)	––	Depression, life satisfaction	Gender Children	Stronger for women^c^ Stronger for couples with children	USA	NSFH
Andreß and Bröckel (2007)	––	Life satisfaction	Gender	Stronger for men	Germany	SOEP
Liu and Umberson (2008)^d^		Self-rated health	Cohort	Stronger for recent cohorts	USA	NHIS
Mandemakers et al. (2010)	––	Psychological distress	SES of origin family	Stronger for low-status persons	UK	NCDS
Monden and Uunk (2013)	0	Self-rated health	Gender	n.s.	Europe	ECHP

^a^A main effect refers to a decline in well-being after divorce.

^b^“Stronger” means a more negative divorce effect; n.s. means no significant interaction. In some instances, two-way interactions could not be interpreted because of the presence of three-way interactions. In these cases, we do not report on the two-way interactions. The same applies to some main effects.

^c^Different interactions were found for other types of outcomes, such as alcohol abuse (see the text).

^d^Main effect not given.

Another moderator variable that has been studied is marital quality. The evidence is mixed, suggesting that leaving an unhappy marriage is not generally better for well-being, in contrast to what relief or escape hypotheses suggest (Johnson and Wu 2002; Kalmijn and Monden 2006; Williams 2003). Interactions with age have also revealed inconsistent patterns (Blekesaune and Barrett 2005; Williams and Umberson 2004). Some evidence suggests that divorce has become more detrimental across marriage cohorts in the United States (Liu and Umberson 2008), but how this has changed elsewhere is not clear.

The presence of children before divorce has been studied only twice to our knowledge (Blekesaune and Barrett 2005; Williams and Dunne-Bryant 2006). Both studies concluded that couples with children suffer more from a divorce than couples without children. In the study by Blekesaune and Barrett (2005), this conclusion was based on register data with an indirect indicator for well-being, measuring the number of times people were officially registered as ill. Williams and Dunne-Bryant (2006) used more direct measures, but their study was based on data that are now relatively old (from the late 1980s and early 1990s). More importantly, that study could analyze only two waves of data—a design that offers limited information about the adjustment process after divorce and the degree to which children moderate this process. Two-wave panel studies also carry the risk that the predivorce measurement is colored by the impending divorce, especially when the divorce occurred shortly after the first wave.

To understand how children could moderate the impact of divorce, we first outline general ideas about how a divorce affects people’s lives: in particular, the crisis model and the resource model. Next, we apply these ideas to our key moderators of interest: namely, the presence and age of children before divorce. We pay special attention to differences between men and women, given that the effects of children on the economic and social costs of divorce are divided along gender lines. To uncover these differences, we consider the moderating effects of children on three types of well-being: satisfaction with life (“general well-being”), satisfaction with income (“economic well-being”), and satisfaction with family life (“family well-being”). We are aware of only one study that has studied divorce in relation to domain-specific well-being outcomes (Andreß and Bröckel 2007). That study, however, was limited to general and economic well-being and did not consider the presence of children.

## The Crisis and Resource Model of Divorce

According to the crisis model (Amato 1993, 2000; Johnson and Wu 2002; McLanahan and Sandefur 1994), a divorce not only is emotionally straining but also brings a series of practical changes, each of which can be stressful. These secondary stressors include moving, adjusting to living alone, making new financial arrangements, dividing consumption goods, informing families and friends, finding a new partner, and so forth. An important tenet of the crisis model is that the effect of divorce is short-lived. After practical arrangements have been made and people are accustomed to new routines, they will start to feel better. Similarly, the emotional impact of losing a partner is believed to be transient (Stroebe et al. 2007). Another expectation of the crisis model is that the decline in well-being begins before separation. Divorce is a process—not an event—and well-being suffers throughout this process.

A second theoretical perspective on how divorce affects well-being is the resource model (Johnson and Wu 2002; McLanahan and Sandefur 1994; Soons et al. 2009).^2^ This model typically distinguishes between economic and social resources. A divorce involves declines in these resources, which in turn involve declines in well-being and health (Andreß and Hummelsheim 2009; McLanahan and Sandefur 1994). Economic resources typically decline after divorce, especially for women (Andreß and Hummelsheim 2009; Poortman 2000; Uunk 2004). This result occurs for four main reasons: (1) men typically contribute a higher share of household income; (2) alimony payments are often insufficient for child maintenance; (3) earning capacities in the presence of children are limited; and (4) economies of scale are lost when couples separate (Holden and Smock 1991). Moving into a smaller home or into a poorer neighborhood are often part of this decline.

Social resources comprise the set of personal relationships and networks on which people can rely. A divorce not only disrupts a primary tie but also involves the risk of losing ties to family members and mutual friends, although there may also be increases in friendship contacts, which compensate for these losses (Terhell et al. 2004). Another example of a decline in social resources is the loss of neighborhood ties when people are forced to move after divorce (Hagan et al. 1996).

## Hypotheses on the Moderating Role of Children

### Presence of Children

How would children moderate the impact of divorce? Our first hypothesis is that children intensify the negative effect of divorce on the general well-being of both men and women (Hypothesis 1). The crisis and resource models offer two main reasons for this expectation. First, the crisis associated with the separation process will be more intense and last longer if children are involved, in particular because couples who divorce with children experience an increase in parenting-related stress (Williams and Dunne-Bryant 2006). Parents and children have to find new living arrangements and new ways of maintaining their relationships. Compared with divorcees without children, personal concerns about children’s well-being may constitute an additional source of stress after separation. The same applies to contact with ex-partners. Divorced parents often have to stay in touch, which can prove another source of strain and continued conflict. In the absence of children, ex-partners more often experience a swift decline in contact (Fischer et al. 2005). Such a “clean break” can be beneficial for recovery in terms of well-being but is less likely to occur for couples with children.

Second, divorce-related declines in resources are also exacerbated by the presence of children. Looking at economic resources, mothers generally find it more difficult to work for pay after divorce than divorced women without children (Van Damme 2010). Moreover, mothers with young children at home are less likely to find a new partner, which further reduces their options for economic and emotional recovery (Dewilde and Uunk 2008; Ivanova et al. 2013). As a result, the economic costs of divorce are higher for mothers than for childless women. This reality is also true when comparing fathers with childless men because divorce often entails long-term commitments in terms of child maintenance and alimony as well as losses in economies of scale. Compared with mothers, however, the child-related increase in the economic costs of divorce for men will be far less pronounced, in particular because breadwinner fathers do not face similar constraints in terms of labor force participation (Poortman 2000).

A contrasting picture emerges when looking at the social costs of divorce. Fathers are at a greater risk of losing day-to-day contact with their children (Juby et al. 2007; Swiss and Le Bourdais 2009), and a substantial minority of divorced fathers never see their children after divorce (Kalmijn 2015). Not all divorced fathers miss their children, but many indicate feelings of dissatisfaction and loss (Parkinson and Smyth 2004). As a result, the social costs of divorce among fathers can be expected to exceed those of childless men.

To summarize, the crisis model implies that the presence of children intensifies the negative effects of divorce on general well-being both among men and women. The resource model is also consistent with this expectation for general well-being but posits differential effects, depending on the type of resource involved. A focus on domain-specific aspects of well-being allows us to separate these two mechanisms. In terms of *economic well-being*, we expect the moderating effect of children to be larger for women than for men (Hypothesis 2a). In terms of *family well-being*, we expect the moderating effect of children to be larger for men than for women (Hypothesis 2b).

### Age of Children

In addition to distinguishing between the presence and absence of children before divorce, we consider how this effect might vary with child age. We posit that the moderating effect of children declines if children are older. Specifically, we expect that the negative effects of divorce on general well-being are largest in the presence of preschool-age children before divorce, and smaller in the presence of older resident children (Hypothesis 3). This expectation is consistent with the factors highlighted by the resource model: the younger the children, the larger the divorce-related declines in economic and social resources. Again, we expect that these effects are divided along gender lines. Regarding economic resources, costs are highest for mothers of preschool-aged children. If children are older, divorced women’s earnings and labor force participation are higher (Van Damme 2010). Regarding social resources, child age influences the strength of the tie to fathers: the younger children are, the less time fathers spend with them (Swiss and Le Bourdais 2009).

These considerations are an extension to the rationale outlined earlier, suggesting that gender differences emerge primarily when looking at domain-specific aspects of well-being. In terms of *economic well-being*, we expect that the moderating effect of child age is larger for mothers than for fathers (Hypothesis 4a). In terms of *family well-being*, we expect that the moderating effect of child age is larger for fathers than for mothers (Hypothesis 4b).

## Adjustment to Divorce

The aforementioned hypotheses do not distinguish between initial declines in well-being and the subsequent adjustment process. According to the set point theory, well-being gradually reverts to predivorce levels. Although this theory posits that people eventually recover, it does not make specific predictions about the speed of the adjustment process (Anusic et al. 2014; Soons et al. 2009). Findings on this process are mixed. Some researchers have reported positive duration effects on well-being after divorce (Dupre and Meadows 2007; Kamp Dush 2013), but others have found no duration effects (Johnson and Wu 2002; Williams and Umberson 2004).

In our study, the question is whether and how the adjustment process may differ depending on the presence and age of children. On the one hand, social costs for fathers may increase over time because they may not be able to maintain the relationships with their children. On the other hand, social costs may decline because children’s feelings of being “caught in the middle” may weaken when they grow older, which may improve contact with nonresident fathers (Amato and Afifi 2006). Opposing predictions can also be made for economic costs. On the one hand, the economic costs for women may decline when children are older because restrictions in terms of employment and remarriage chances decline (Ivanova et al. 2013). On the other hand, economic costs may be stable because women who are unemployed after divorce may not be able to reenter the labor market because of the loss of work experience and a depreciation of their human capital (Van Damme 2010).

## Sources of Bias

When estimating the moderating effect of children, researchers must consider several potential sources of bias. First, research has shown that children raise the threshold to divorce (De Graaf and Kalmijn 2006). If couples still divorce, this event is likely to indicate more intense conflict and more serious marital problems compared with childless divorcees. Given that there is less room for a decline in well-being between waves when well-being is low to begin with—a phenomenon called “floor effects” in panel data (Wang et al. 2008)—post-divorce declines in well-being may be less pronounced if couples have children, which could potentially suppress the moderator effects that we hypothesized. In the present study, we therefore consider differences between childless couples and couples with children in their predivorce levels of well-being.

A second potential source of bias lies in marital duration. Within the age range that we study, most childless couples are married for a shorter period. If effects of divorce on well-being are smaller when marriages were shorter, this could lead to a spurious interaction effect. We control for this by considering the age of the married respondent at divorce as a proxy for marital duration. Third, we also consider differences along socioeconomic lines. Initial analyses of our data suggested that lower socioeconomic groups are more likely to divorce in the presence of preschool-aged children. This could bias our interaction effect if the impact of divorce differs between socioeconomic groups.

## Data and Method

### Data

Our analysis was based on data from 29 waves of the SOEP (Version 29, 2013, doi:10.5684/soep.v29), a household panel survey in which each household member age 17 and older is interviewed separately (for detailed information, see Wagner et al. 2007).

For our purposes, these data yielded three benefits. First, the large sample size allowed us not only to examine average effects of divorce but also to test for heterogeneity in these effects on well-being. Second, the large window of closely spaced observations was ideally suited to track short-term and long-term changes in well-being across the divorce process. Third, the SOEP offers a comprehensive set of indicators for well-being, enabling us to test our theoretical considerations about differential effects of divorce on general well-being, economic well-being, and family well-being. We restricted the analytical sample to persons who divorced across their observation period in the panel. To capture this transition, we selected 2,353 respondents for whom we observed a transition from a marital union to divorce.^3^ If a person divorced more than once, we examined the first divorce recorded in the panel.

We identified a divorce by a change of marital status from “married and living together” to “divorced.” Because a change in the legal status from married to divorced may involve some delay (resulting from an obligatory year of separation), we also assigned the status of divorced if a respondent reported a change from “married and living together” to “married but separated.” Hence, our definition of divorce captures the year of separation. We did not consider unmarried cohabitation or unions that remained unmarried. Given that in the German context of our study, the large majority of couples who have children are married, comparisons with unmarried couples or observations of unmarried unions would seem less relevant.

To estimate predivorce levels of well-being, we considered all panel observations up to one year before divorce.^4^ We also considered all observations after divorce to estimate short-term and long-term patterns of adaptation, including the post-divorce observations in which persons began living with a new partner. Although repartnering affects well-being (Sweeney 2010), the chances of finding a new partner are affected negatively by having children (Ivanova et al. 2013). Hence, repartnering may be a mediator of our moderator effects of interest, and not a confounder. After all restrictions, the analytical sample consisted of 2,353 individuals with 35,146 panel observations (i.e., person-years). The average number of annual observations per respondent was 15 years, with a range of 2–28.^5^

### Measures

#### Outcome Variables

To test our hypotheses about changes in different domains of well-being, we used three outcome variables. Our measures of *general well-being* and *economic well-being* were based on the survey questions, “How satisfied are you with your life, all things considered?” and “How satisfied are you with your household income?” Data on these measures were available at all 29 panel waves conducted between 1984 and 2012. Our measure for *family well-being* was based on the survey question, “How satisfied are you with your family life?” This question allowed respondents to define “family” as they wished, given that no further specification was offered. Data on this measure were available annually since 2006. Thus, our analyses of family well-being were based on panel observations from 2006 onward. Marital unions observed before 2006 were still included in this subset. Each outcome variable was measured on an 11-point Likert scale ranging from 0 (“completely dissatisfied”) to 10 (“completely satisfied”). Table 2 shows descriptive statistics for the outcome variables.

**Table 2:** Descriptive statistics for outcome variables

	Full Sample^a^			Analytic Sample^b^		
	*M*	SD	*N*	*M*	SD	*N*
Satisfaction With Life						
Overall	6.98	1.81	352,259	6.65	1.94	35,056
Between		1.49	45,226		1.32	2,353
Within		1.28			1.46	
Satisfaction With Income						
Overall	6.16	2.32	346,398	5.74	2.44	34,776
Between		1.99	44,852		1.70	2,353
Within		1.59			1.83	
Satisfaction With Family Life						
Overall	7.69	1.97	89,645	6.94	2.39	8,271
Between		1.68	23,051		1.93	1,595
Within		1.21			1.57	

*Source*: German Socio-Economic Panel Study 1984–2012, release 2013.

^a^Including all observations up to age 60. Excluding high-income sample G.

^b^Analytic sample restricted to individuals observed across the transition from “married and living together” to “divorced.”

#### Divorce Variables

To assess the short-term and long-term impact of divorce on these outcomes, we used three variables: (1) a dummy variable changing from 0 in all predivorce observations to 1 in all postdivorce observations; (2) a linear duration variable counting the years after divorce, starting from 0 in the wave in which a transition to divorce was observed; and (3) a squared duration variable. These measures jointly represented the effect of divorce on the outcomes, allowing us to study the initial impact as well as long-term patterns of adaptation: the dummy variable captured initial drops in well-being (i.e., duration variables equaling 0). The duration variables captured linear and curvilinear adaptation. We found this functional form to be an adequate and parsimonious specification after assessing changes in the outcomes based on a set of dummy variables that allowed for year-to-year changes in the effects of divorce on the outcome variables.

#### Moderator Variables

We interacted the divorce variables with an indicator variable for whether *at least one child under 18* was living in the respondent’s household in the year before divorce (0 = no, 1 = yes) to test our hypotheses about heterogeneity in the effects of divorce; people in the “no” category were childless, had no resident children (e.g., empty nest), or had no children younger than 18 years. To assess gender differences in the moderating effects of children, we added further interaction terms between the divorce variables and gender (0 = female, 1 = male) and three-way interactions between the divorce variables, the child indicator, and gender. In a final step, we replaced the child indicator by a set of indicator variables for the *age of the youngest child* living in the respondent’s household in the year before divorce (0–4 years, 5–12 years, 13–18 years, or no children under 18). All child variables were based on the situation before the divorce and did not vary over time, given our use of interactions with the divorce variables.

Using this analytical setup, we relied on time since the divorce as the dynamic element in the model. For two reasons, we did not include children’s ages (or the youngest child’s age) as further dynamic elements. First, our interest was in how child age at divorce moderated divorce effects rather than in the effects of changes in child age on parental well-being. Second, a time-varying variable for the age of the youngest child would be collinear with the variable for time since divorce, which was already included in our models.

Information about custody was incomplete. Research on 14-year-old school children from divorced families in Germany in 2010–2011 showed that 71 % lived with the mother, 13 % lived with the father, 10 % had a co-parenting arrangement, and 7 % lived with neither parent (Kalmijn 2015). In our SOEP data, the children are, on average, younger so that the percentages living with the mother after divorce are higher (Westphal et al. 2014).

#### Controls

We controlled for potential confounders in the relationship between the presence of and age of children before divorce and the consequences of divorce for well-being. These include age at divorce, education (reference = less than secondary degree), immigrant status (reference = native German), and East German (reference = West German).^6^ We centered all these controls on their sample means. Furthermore, we included a control for the calendar year of divorce (centered on 2010) in the models for general and economic well-being. In contrast to family well-being (measured from 2006 until 2012), these outcome variables spanned an observation period of almost three decades (1984 until 2012). Our control for calendar year ensured that divorce effects were conditioned on a comparable period of time across all outcomes. In all models, we interacted the time-constant controls with the divorce variables. Finally, we included a measure of age at divorce as a proxy for marital duration.

Table 3 gives an overview of the moderator and control variables. Table 4 shows how the sociodemographic characteristics included as control variables varied by our key moderators of interest: namely, the presence and age of children before divorce.

**Table 3:** Descriptive statistics for moderator and control variables (*N* = 2,353)

	*M*	SD	Min.	Max.
Moderator Variables				
Children before divorce^a^				
None	.35	.48	0	1
Age 0–4	.21	.41	0	1
Age 5–12	.27	.45	0	1
Age 13–18	.16	.37	0	1
Male	.46	.50	0	1
Control Variables				
Calendar year of divorce	1999.53	7.39	1985	2012
Age at divorce	38.42	9.03	19	60
Education^b^				
Low	.38	.49	0	1
Mid	.45	.50	0	1
High	.17	.37	0	1
East German^c^	.23	.42	0	1
Immigrant^d^	.19	.39	0	1

*Source*: German Socio-Economic Panel Study 1984–2012, release 2013.

^a^Children living in the respondent’s household in the year before divorce; age refers to the age of the youngest child.

^b^Low education = up to lower secondary vocational degree (CASMIN 1a–c). Mid education = up to higher secondary degree plus vocational training (CASMIN 2a–c). High education = lower and higher tertiary degree (CASMIN 3a–b).

^c^Living in East Germany (former German Democratic Republic) in 1989.

^d^First-generation and second-generation immigrant.

**Table 4:** Sociodemographic correlates of having children before divorce (*N* = 2,353)

				Education^b^				
Children Before Divorce^a^	Male	Calendar Year of Divorce	Age at Divorce	Low	Mid	High	East German^c^	Immigrant^d^
None	0.49	1999.24	39.53	0.36	0.44	0.20	0.19	0.20
Age 0–4	0.43	1998.80	32.45	0.43	0.44	0.13	0.20	0.22
Age 5–12	0.45	1999.62	38.13	0.41	0.46	0.14	0.24	0.19
Age 13–18	0.45	2000.97	44.36	0.32	0.48	0.20	0.33	0.15
Total	0.46	1999.53	38.42	0.38	0.45	0.17	0.23	0.19

*Source*: German Socio-Economic Panel Study 1984–2012, release 2013.

^a^Children living in the respondent’s household in the year before divorce; age refers to the age of the youngest child.

^b^Low education = up to lower secondary vocational degree (CASMIN 1a–c). Mid education = up to higher secondary degree plus vocational training (CASMIN 2a–c). High education = lower and higher tertiary degree (CASMIN 3a–b).

^c^Living in East Germany (former German Democratic Republic) in 1989.

^d^First-generation and second-generation immigrant.

## Models

We estimated random-effects hierarchical linear models for annual panel observations nested in persons. Because every respondent in the sample experienced a divorce across the observation period, there was no risk that the event indicators were correlated with unmeasured, time-constant characteristics (Allison 1994). As a result, the bias-reducing properties of the fixed-effects estimator did not apply. In this case, the random-effects generalized least squares estimator is preferable because it (1) is more efficient and (2) allows for the inclusion of main effects for time-constant variables. The second benefit was particularly important for our purposes, given that predivorce well-being may differ between childless couples and couples with children. As noted, predivorce well-being may be lower in the latter group, introducing potential floor effects in the estimation of further declines in well-being.

We estimated three models for each outcome. Model 1 included only the divorce variables. In Model 2, we interacted the divorce variables with the indicator for the presence of children. In Model 3, we added a three-way interaction with gender to test whether the interactions between divorce and the presence of children varied between men and women. All models included control variables as well as interactions between time-constant controls and the divorce variables. Given that all control variables were centered, these extra interactions did not affect the key interaction effects pertaining to our hypotheses.

**Fig. 1:**
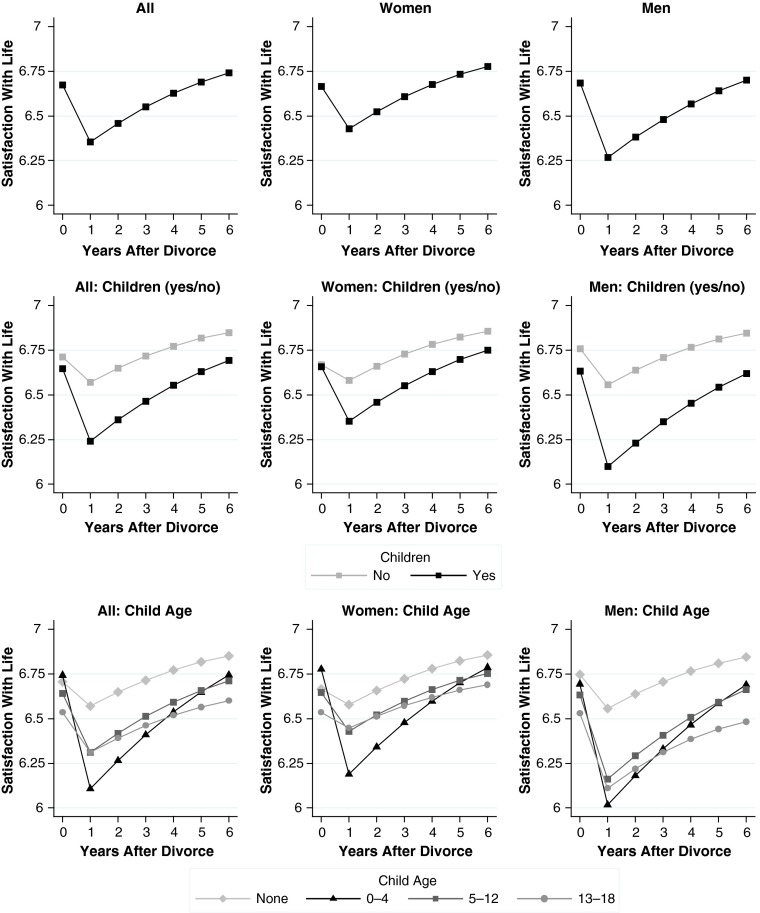
Changes in psychological well-being. See Tables 2 and 3 for details on the measures. Source: German Socio-Economic Panel Study 1984–2012, release 2013

**Fig. 2:**
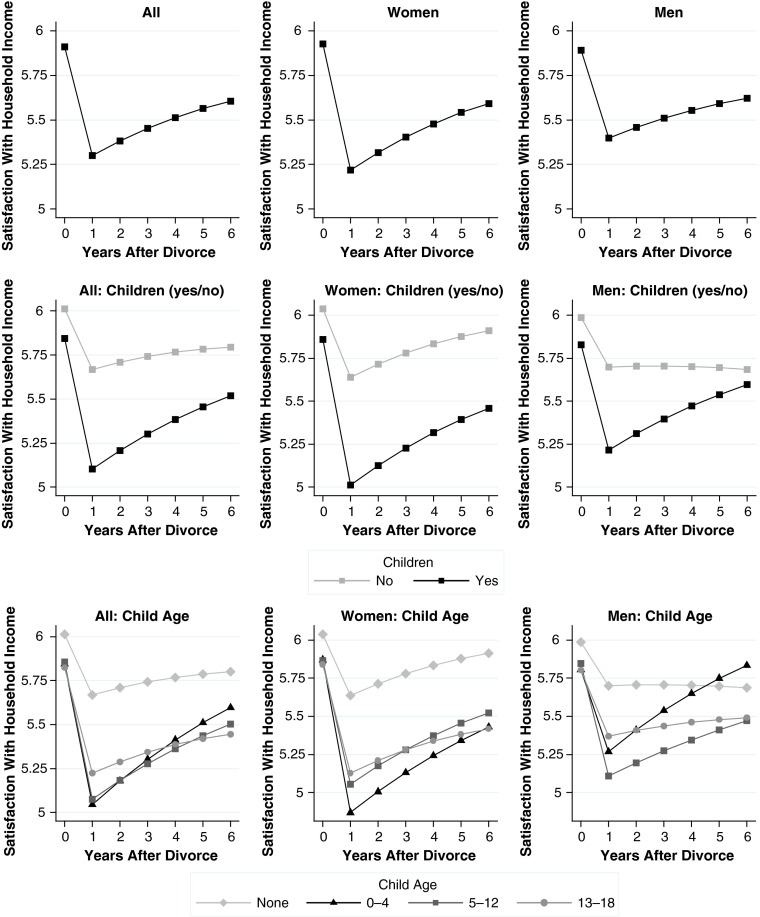
Changes in economic well-being. See Tables 2 and 3 for details on the measures. Source: German Socio-Economic Panel Study 1984–2012, release 2013

**Fig. 3:**
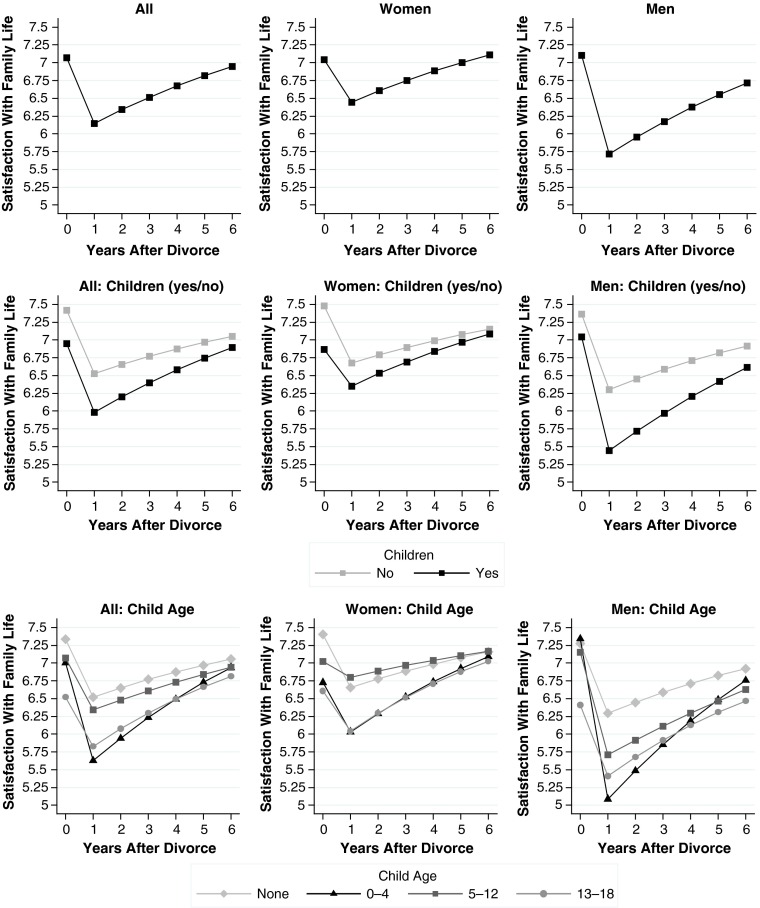
Changes in family well-being. See Tables 2 and 3 for details on the measures. Source: German Socio-Economic Panel Study 1984–2012, release 2013

Results for all models are shown in Table 5 (general well-being), Table 6 (economic well-being), and Table 7 (family well-being). We plot our main findings from these models in Fig. 1 (general well-being), Fig. 2 (economic well-being), and Fig. 3 (family well-being). Each figure comprises nine plots: the top row shows overall effects, the middle row shows moderator effects of the binary child indicator, and the bottom row shows moderator effects of the indicator for child age. The left column shows effects for men and women combined, the middle column shows effects for women, and the right column shows effects for men. We present the results for child age only in the plots so that the tables are easier to read.

**Table 5:** Random-effects linear regression models for change in general well-being

	Model 1: Divorce		Model 2: Divorce × Children		Model 3: Divorce × Children × Gender	
Divorce (ref. = >1 year before)^a^						
Divorce	–0.477**	(0.054)	–0.261**	(0.066)	–0.211**	(0.077)
Duration	0.135**	(0.019)	0.107**	(0.021)	0.107**	(0.023)
Duration, squared	–0.008**	(0.001)	–0.008**	(0.002)	–0.008**	(0.002)
Children (ref. = no)^b^						
Yes			–0.063	(0.059)	–0.009	(0.081)
Gender (ref. = female)						
Male					0.089	(0.095)
Children × Gender						
Children × Male					–0.116	(0.117)
Divorce × Children						
Divorce × Children			–0.305**	(0.054)	–0.248**	(0.074)
Duration × Children			0.042**	(0.013)	0.030^†^	(0.018)
Duration, squared × Children			–0.001	(0.001)	–0.001	(0.001)
Divorce × Gender						
Divorce × Male					–0.119	(0.087)
Duration × Male					0.004	(0.021)
Duration, squared × Male					–0.000	(0.001)
Divorce × Children × Gender						
Divorce × Children × Male					–0.135	(0.107)
Duration × Children × Male					0.022	(0.026)
Duration, squared × Children × Male					0.000	(0.001)
Controls						
Age at divorce^c^	0.015**	(0.004)	0.015**	(0.004)	0.015**	(0.004)
Divorce × Age at divorce	–0.006	(0.004)	–0.007^†^	(0.004)	–0.005	(0.004)
Duration × Age at divorce	0.001	(0.001)	0.002^†^	(0.001)	0.001	(0.001)
Duration, squared × Age at divorce	–0.000**	(0.000)	–0.000**	(0.000)	–0.000**	(0.000)
Year of divorce^d^	0.012**	(0.004)	0.012**	(0.004)	0.012**	(0.004)
Divorce × Year of divorce	–0.004	(0.004)	–0.003	(0.004)	–0.003	(0.004)
Duration × Year of divorce	0.001	(0.001)	0.001	(0.001)	0.001	(0.001)
Duration, squared × Year of divorce	–0.000*	(0.000)	–0.000*	(0.000)	–0.000*	(0.000)
Education (ref. = low)^e^						
Intermediate	0.154*	(0.065)	0.148*	(0.065)	0.150*	(0.066)
High	0.372**	(0.084)	0.362**	(0.084)	0.362**	(0.084)
Divorce × Intermediate	0.125*	(0.059)	0.106^†^	(0.060)	0.092	(0.060)
Divorce × High	0.155*	(0.076)	0.122	(0.076)	0.120	(0.076)
Duration × Intermediate	0.012	(0.014)	0.015	(0.014)	0.015	(0.015)
Duration × High	0.040*	(0.019)	0.044*	(0.019)	0.043*	(0.019)
Duration, squared × Intermediate	–0.001	(0.001)	–0.001	(0.001)	–0.001	(0.001)
Duration, squared × High	–0.001	(0.001)	–0.001	(0.001)	–0.001	(0.001)
Immigrant (ref. = native German)^f^	–0.210**	(0.073)	–0.207**	(0.073)	–0.206**	(0.073)
Divorce × Immigrant	–0.047	(0.067)	–0.057	(0.066)	–0.065	(0.067)
Duration × Immigrant	–0.041*	(0.017)	–0.040*	(0.017)	–0.040*	(0.017)
Duration, squared × Immigrant	0.002*	(0.001)	0.002^†^	(0.001)	0.002*	(0.001)
East German (ref. = West)^g^	–0.882**	(0.071)	–0.874**	(0.071)	–0.874**	(0.071)
Divorce × East German	0.306**	(0.066)	0.334**	(0.067)	0.331**	(0.067)
Duration × East German	–0.029	(0.019)	–0.032^†^	(0.019)	–0.031	(0.019)
Duration, squared × East German	0.003*	(0.001)	0.002*	(0.001)	0.002*	(0.001)
Constant	6.601**	(0.066)	6.656**	(0.077)	6.618**	(0.089)
Number of Observations	34,954		34,954		34,954	

*Source*: German Socio-Economic Panel Study 1984–2012, release 2013.

^a^Reference category comprises all observations up to one year before divorce; divorce is an indicator variable for the year of divorce; duration variables count the years after divorce (0 in the year of divorce).

^b^At least one child living in the respondent’s household in the year before divorce.

^c^Centered on the mean.

^d^Centered on 2010.

^e^Low education = up to lower secondary vocational degree (CASMIN 1a–c), intermediate education = up to higher secondary degree plus vocational training (CASMIN 2a–c), high education = lower and higher tertiary degree (CASMIN 3a–b); centered on the mean.

^f^First-generation or second-generation immigrant; centered on the mean.

^g^Living in East Germany (former German Democratic Republic) in 1989; centered on the mean. All models control for age in three-yearly intervals.

^†^ *p* < .10; *p < .05; **p < .01

**Table 6:** Random-effects linear regression models for change in economic well-being

	Model 4: Divorce		Model 5: Divorce × Children		Model 6: Divorce × Children × Gender	
Divorce (ref. = >1 year before)^a^						
Divorce	–0.769**	(0.068)	–0.439**	(0.083)	–0.524**	(0.098)
Duration	0.118**	(0.023)	0.070**	(0.026)	0.111**	(0.029)
Duration, squared	–0.007**	(0.002)	–0.006**	(0.002)	–0.008**	(0.002)
Children (ref. = no)^b^						
Yes			–0.167*	(0.076)	–0.178^†^	(0.104)
Gender (ref. = female)						
Male					–0.050	(0.121)
Children × Gender						
Children × Male					0.019	(0.149)
Divorce × Children						
Divorce × Children			–0.466**	(0.068)	–0.487**	(0.093)
Duration × Children			0.070**	(0.017)	0.039^†^	(0.023)
Duration, squared × Children			–0.002^†^	(0.001)	–0.001	(0.001)
Divorce × Gender						
Divorce × Male					0.187^†^	(0.110)
Duration × Male					–0.082**	(0.027)
Duration, squared × Male					0.004*	(0.001)
Divorce × Children × Gender						
Divorce × Children × Male					0.070	(0.135)
Duration × Children × Male					0.058^†^	(0.033)
Duration, squared × Children × Male					–0.002	(0.002)
Controls						
Age at divorce^c^	0.024**	(0.005)	0.023**	(0.005)	0.024**	(0.005)
Divorce × Age at divorce	–0.011*	(0.004)	–0.012**	(0.004)	–0.014**	(0.004)
Duration × Age at divorce	–0.002*	(0.001)	–0.001	(0.001)	–0.001	(0.001)
Duration, squared × Age at divorce	0.000	(0.000)	–0.000	(0.000)	–0.000	(0.000)
Year of divorce^d^	–0.005	(0.005)	–0.005	(0.005)	–0.005	(0.005)
Divorce × Year of divorce	–0.006	(0.005)	–0.004	(0.005)	–0.003	(0.005)
Duration × Year of divorce	0.002	(0.001)	0.002	(0.001)	0.002	(0.001)
Duration, squared × Year of divorce	–0.000*	(0.000)	–0.000*	(0.000)	–0.000*	(0.000)
Education (ref. = low)^e^						
Intermediate	0.410**	(0.084)	0.397**	(0.083)	0.395**	(0.084)
High	0.858**	(0.107)	0.835**	(0.107)	0.833**	(0.107)
Divorce × Intermediate	0.124^†^	(0.075)	0.093	(0.075)	0.109	(0.075)
Divorce × High	0.200*	(0.096)	0.149	(0.096)	0.154	(0.096)
Duration × Intermediate	0.028	(0.018)	0.033^†^	(0.018)	0.029	(0.018)
Duration × High	0.054*	(0.024)	0.061*	(0.024)	0.061*	(0.024)
Duration, squared × Intermediate	–0.002^†^	(0.001)	–0.002^†^	(0.001)	–0.001	(0.001)
Duration, squared × High	–0.001	(0.001)	–0.002	(0.001)	–0.001	(0.001)
Immigrant (ref. = native German)^f^	–0.364**	(0.093)	–0.360**	(0.093)	–0.359**	(0.093)
Divorce × Immigrant	0.093	(0.084)	0.075	(0.084)	0.077	(0.084)
Duration × Immigrant	–0.133**	(0.021)	–0.130**	(0.021)	–0.129**	(0.021)
Duration, squared × Immigrant	0.006**	(0.001)	0.005**	(0.001)	0.005**	(0.001)
East German (ref. = West)^g^	–1.226**	(0.091)	–1.207**	(0.091)	–1.206**	(0.091)
Divorce × East German	0.379**	(0.083)	0.423**	(0.084)	0.423**	(0.084)
Duration × East German	–0.050*	(0.024)	–0.054*	(0.024)	–0.054*	(0.024)
Duration, squared × East German	0.003*	(0.001)	0.003*	(0.002)	0.003^†^	(0.002)
Constant	5.793**	(0.084)	5.923**	(0.098)	5.946**	(0.113)
Number of Observations	34,672		34,672		34,672	

*Source*: German Socio-Economic Panel Study 1984–2012, release 2013.

^a^Reference category comprises all observations up to one year before divorce; divorce is an indicator variable for the year of divorce; duration variables count the years after divorce (0 in the year of divorce).

^b^At least one child living in the respondent’s household in the year before divorce.

^c^Centered on the mean.

^d^Centered on 2010.

^e^Low education = up to lower secondary vocational degree (CASMIN 1a–c), intermediate education = up to higher secondary degree plus vocational training (CASMIN 2a–c), high education = lower and higher tertiary degree (CASMIN 3a–b); centered at the mean.

^f^First-generation or second-generation immigrant; centered on the mean.

^g^Living in East Germany (former German Democratic Republic) in 1989; centered on the mean. All models control for age in three-yearly intervals.

^†^ *p* < .10; *p < .05; **p < .01

**Table 7:** Random-effects linear regression models for change in family well-being

	Model 7: Divorce		Model 8: Divorce × Children		Model 9: Divorce × Children × Gender	
Divorce (ref. = >1 year before)^a^						
Divorce	–1.140**	(0.116)	–1.030**	(0.168)	–0.926**	(0.213)
Duration	0.216**	(0.029)	0.142**	(0.038)	0.127**	(0.046)
Duration, squared	–0.008**	(0.001)	–0.005**	(0.002)	–0.005*	(0.002)
Children (ref. = no)^b^						
Yes			–0.464*	(0.197)	–0.614*	(0.261)
Gender (ref. = female)						
Male					–0.120	(0.304)
Children × Gender						
Children × Male					0.298	(0.379)
Divorce × Children						
Divorce × Children			–0.184	(0.184)	0.221	(0.246)
Duration × Children			0.102**	(0.036)	0.073	(0.047)
Duration, squared × Children			–0.003*	(0.002)	–0.003	(0.002)
Divorce × Gender						
Divorce × Male					–0.291	(0.289)
Duration × Male					0.040	(0.057)
Duration, squared × Male					–0.002	(0.003)
Divorce × Children × Gender						
Divorce × Children × Male					–0.899*	(0.360)
Duration × Children × Male					0.062	(0.071)
Duration, squared × Children × Male					0.000	(0.003)
Controls						
Age at divorce^c^	–0.003	(0.025)	–0.011	(0.025)	–0.012	(0.025)
Divorce × Age at divorce	–0.008	(0.012)	–0.007	(0.012)	0.002	(0.012)
Duration × Age at divorce	0.004^†^	(0.002)	0.005*	(0.002)	0.004^†^	(0.002)
Duration, squared × Age at divorce	–0.000*	(0.000)	–0.000*	(0.000)	–0.000*	(0.000)
Year of divorce^d^						
Divorce × Year of divorce	0.389^†^	(0.224)	0.350	(0.224)	0.426^†^	(0.224)
Duration × Year of divorce	0.258	(0.270)	0.199	(0.272)	0.211	(0.271)
Duration, squared × Year of divorce	–0.332	(0.212)	–0.344	(0.212)	–0.464*	(0.213)
Education (ref. = low)^e^	–0.024	(0.254)	–0.036	(0.256)	–0.045	(0.256)
Intermediate	0.056	(0.039)	0.064	(0.039)	0.068^†^	(0.039)
High	0.028	(0.050)	0.038	(0.050)	0.037	(0.050)
Divorce × Intermediate	–0.003	(0.002)	–0.003	(0.002)	–0.003	(0.002)
Divorce × High	–0.003	(0.002)	–0.003	(0.002)	–0.003	(0.002)
Duration × Intermediate	–0.313	(0.235)	–0.277	(0.235)	–0.277	(0.234)
Duration × High	–0.371^†^	(0.222)	–0.353	(0.222)	–0.371^†^	(0.222)
Duration, squared × Intermediate	0.008	(0.046)	0.001	(0.046)	–0.002	(0.046)
Duration, squared × High	0.001	(0.002)	0.001	(0.002)	0.001	(0.002)
Immigrant (ref. = native German)^f^	–0.466*	(0.236)	–0.459^†^	(0.236)	–0.479*	(0.235)
Divorce × Immigrant	0.291	(0.225)	0.295	(0.225)	0.283	(0.225)
Duration × Immigrant	–0.030	(0.045)	–0.032	(0.045)	–0.027	(0.045)
Duration, squared × Immigrant	0.000	(0.002)	0.000	(0.002)	–0.000	(0.002)
East German (ref. = West)^g^	–0.466*	(0.236)	–0.459^†^	(0.236)	–0.479*	(0.235)
Divorce × East German	0.291	(0.225)	0.295	(0.225)	0.283	(0.225)
Duration × East German	–0.030	(0.045)	–0.032	(0.045)	–0.027	(0.045)
Duration, squared × East German	0.000	(0.002)	0.000	(0.002)	–0.000	(0.002)
Constant	6.914**	(0.209)	7.299**	(0.254)	7.366**	(0.289)
Number of Observations	8,244		8,244		8,244	

*Source*: German Socio-Economic Panel Study 1984–2012, release 2013.

^a^Reference category comprises all observations up to one year before divorce; divorce is an indicator variable for the year of divorce; duration variables count the years after divorce (0 in the year of divorce).

^b^At least one child living in the respondent’s household in the year before divorce.

^c^Centered on the mean.

^d^Centered on 2010.

^e^Low education = up to lower secondary vocational degree (CASMIN 1a–c), intermediate education = up to higher secondary degree plus vocational training (CASMIN 2a–c), high education = lower and higher tertiary degree (CASMIN 3a–b); centered on the mean.

^f^First-generation or second-generation immigrant; centered on the mean.

^g^Living in East Germany (former German Democratic Republic) in 1989; centered on the mean. All models control for age in three-yearly intervals.

^†^ *p* < .10; *p < .05; **p < .01

## Results for General Well-being

Model 1 in Table 5 presents baseline estimates for changes in general well-being following a divorce. The main effects of the divorce variables represent the average impact of divorce, conditioned on a divorce year of 2010 and mean values on all other time-constant controls. These estimates were in line with previous research: the initial decline amounted to more than one-third of a standard deviation of within-person change in general well-being over time (−0.477 / 1.28 = 0.37). The duration effect was positive, indicating adjustment. Figure 1 shows that in the years after divorce, people fully recovered from this initial decline in well-being (Fig. 1, top-left plot).

Turning to heterogeneity behind these average effects, we first hypothesized that children intensify the negative consequences of divorce for general well-being (Hypothesis 1). The findings from Model 2 (Table 5) support this hypothesis. In this model, the main effects of divorce were defined for persons without children in the year before divorce; the interaction effects indicated whether these effects differed for those with at least one resident child in the year before divorce. Results showed that the initial decline in well-being more than doubled in the presence of children (an interaction of −0.305 vs. a main effect of –0.261). Furthermore, the main effect of duration was significantly stronger for people with children than for people without children. As illustrated by the middle plot of Fig. 1, sharper initial declines in general well-being were followed by faster recovery. As a result, the gap to divorcees without children narrowed over time.

In an extension to our initial hypothesis, we expected a gradient by child age: the younger the children before divorce, the larger their parents’ declines in general well-being (Hypothesis 3). The bottom-left plot of Fig. 3 is broadly consistent with this expectation, indicating the largest drops in the presence of preschool-aged children. However, two qualifications apply. First, we found no systematic differences between older age groups; second, the remaining gaps by child age vanished in the post-divorce years, as divorcees with preschool-aged children adapted faster.

In Model 3 (Table 5), we tested whether the moderating effects of children on general well-being differed between men and women. The point estimates for the three-way interactions among the divorce variables, the child indicator, and gender were insignificant. Yet, the direction of the interaction suggests that the moderator effect was somewhat more negative for men—a tendency that could also be seen in the middle and right columns of Fig. 1. Hence, the negative impact of children appeared to be larger for men than for women.

## Results for Domain-Specific Well-being

Hypotheses 2 and 4 were based on the argument that domain-specific measures of well-being are needed to uncover gender differences in the moderating effects of children. To test these hypotheses, we turn to the models for economic well-being (Table 6, Fig. 2) and family well-being (Table 7, Fig. 3). For ease of comparison, models and plots for both outcomes are aligned with those presented for general well-being. To test our remaining hypotheses, looking at the estimates and plots that are based on the full models is sufficient (Models 6 and 9).

Tables 6 and 7 show that a divorce was associated with substantial declines in both domains of well-being (Models 4 and 7). Both types of well-being revealed a positive duration effect and a small negative effect of the squared duration term, indicating adaptation in economic well-being and family well-being during the post-divorce period. The initial effect of divorce was stronger on economic well-being than on family well-being. When we look at men and women combined (left columns of Figs. 2 and 3), we see that children moderated the impact of divorce in the expected direction: for economic well-being (Model 5, Table 6), the negative effect of divorce doubled in the presence of children (an interaction of −0.466 vs. a main effect of −0.439). For family well-being (Model 8, Table 7), this interaction was negative as well, albeit smaller and not statistically significant.

The primary goal of separating the two domains of well-being was to examine gender interactions, which are presented in Models 6 and 9. In terms of economic well-being, we expected that the moderating effects of children would be larger for women than for men (Hypothesis 2a). In terms of family well-being, we expected that the moderating effects of children would be larger for men than for women (Hypothesis 2b). Model 6 for economic well-being shows that the three-way interaction of gender, divorce, and children pointed in the expected direction. The effect, however, was small and not significant (*b* = 0.070, *p* > .05, Table 6). This result is inconsistent with Hypothesis 2a. Looking at Model 9 for family well-being, a much clearer pattern emerged for the corresponding three-way interaction (*b* = −0.899, *p* < .01, Table 7). For women, the moderator effect of children was positive (*b* = 0.221) but not different from 0 at conventional levels of statistical significance. For men, the moderator effect of children was strongly negative (*b* = 0.221 – 0.899 = −0.678). Hence, declines in family well-being after divorce were larger when couples had children, but this was true for men only. This finding is in line with Hypothesis 2b.

Turning to more detailed results broken down by child age, we found support for our remaining hypotheses. In the presence of preschool age children, women’s economic well-being declined by a full scale point in the year of divorce, and this was a stronger drop than was found for men (Fig. 2, bottom row). Although this difference was not significant, the direction was in line with our hypothesis. For family well-being, the plots by child age (bottom row of Fig. 3) are consistent with Hypothesis 4b: the steepest declines in men’s family well-being were found if children were of younger age (Fig. 3, bottom-right plot). In this case, men dropped by almost 2.5 scale points or approximately 2 standard deviations of within-person change in family well-being over time. In contrast to our findings on women’s economic well-being, however, men recovered from these disproportionate declines in family well-being. Six years after divorce, differences compared with childless men had largely disappeared.

## Conclusion and Discussion

According to theoretical models of the divorce process, the presence of children intensifies both the emotional crisis associated with marital breakup and former partners’ loss of economic and social resources. Yet, although these models suggest that divorce is more painful for couples with children, little is known about the role of children as a moderator of divorce effects on well-being. This study addressed this gap of research using long-term panel data from Germany. Following individuals over several years before and after divorce, we investigated whether the presence of children aggravated the impact of divorce on well-being, we assessed the importance of child age, and we tested how men and women differed in this respect. Our empirical analyses went beyond previous work by strengthening the dynamic nature of the analysis and by examining changes in multiple domains of well-being.

Three central findings emerged from the analysis. First, declines in general well-being, economic well-being, and family well-being were sharper if dependent children were present before divorce. Moreover, moderator effects of children tended to be larger if children were younger. In the absence of children, effects of divorce on well-being were trivial. Second, these effects were largely similar among men and women when looking at a general measure of well-being, but domain-specific measures revealed important gender differences. Mothers tended to suffer more in terms of economic well-being—in particular, if preschool-aged children were involved—whereas fathers suffered more in terms of family well-being, although fathers also experienced somewhat larger declines in economic well-being in the presence of children. Third, with the exception of mothers’ economic well-being, the gaps compared with childless divorcees narrowed over time. Moderator effects of children emerged most clearly in the year after divorce, declined across subsequent observations, and vanished six years after separation.

Although these findings are broadly consistent with two earlier studies on this subject (Blekesaune and Barrett 2005; Williams and Dunne-Bryant 2006), they offer novel insight into how children moderate the effect of divorce on well-being. Most notably, we have shown that these moderator effects run through economic and social pathways that are sharply divided along gender lines. Moreover, our findings demonstrate that most of the disproportionate declines in the well-being of divorced parents do not persist. Although the divorce effect is larger for parents, they tend to adapt in the long term.

This study contributes to an emerging line of research on heterogeneity in the effects of divorce on outcomes in adults and children, as summarized in Table 1. With regard to adult outcomes, gender has been the most frequently studied moderator. Most of these studies have found no substantial gender differences in the consequences of divorce for health and well-being, although men and women may adapt on different time scales (Andreß and Bröckel 2007). Our findings suggest that one reason for not finding gender differences in general measures of well-being is that divorce effects are domain-specific particularly when children are involved. Under these conditions, the consequences of divorce for adult well-being are strongly gendered, but specific measures of well-being are required to uncover these differences in the economic and social costs of divorce.

Our finding that fathers suffered most in terms of non-economic outcomes may be related to the fact that women more often initiate a divorce than men (Kalmijn and Poortman 2006). Some fathers may be caught by surprise and therefore suffer more in the social domain, just like some mothers may underestimate the economic consequences of divorce when taking initiative to break up. The need for specific measures to capture gender differences has already been demonstrated in previous studies of men’s and women’s behavioral responses to the divorce crisis: men displayed externalizing behavior in the form of increased alcohol use, whereas women internalized problems in the form of increased depressive symptoms (Simon 2002). We show that men and women may respond to a divorce in different ways and also for different reasons.

A further theoretical implication of our study concerns the linkages among marriage, divorce, and health. The effects of divorce on mental health have been interpreted as evidence that marriage benefits health (Waite and Gallagher 2000). Our study casts doubt on this interpretation given that the negative effects of a marital breakup were generally small for childless people. And because couples with children suffered much more, our results point to the importance of loss and crisis following a divorce rather than to the role of health protection (Johnson and Wu 2002; Pearlin 2009).

Our findings also have implications for the study of child outcomes. The present analysis has shown that although divorce is a powerful stressor per se, the associated crisis is more intense among parents. These findings on children magnifying the negative consequences of divorce support theoretical ideas that adverse outcomes for children are partly transmitted through increased parental stress experienced across the divorce process. Because parental declines in emotional well-being entail strong and direct effects on children’s well-being (Kiernan and Huerta 2008), we posit that declines in parental well-being constitute a major pathway through which divorce affects children. In this regard, an interesting line of speculation is that not divorce per se, but rather parents’ response to it, constitutes one of the main problems in these intergenerational effects. Although further research is needed to substantiate this claim, it calls for a shift in emphasis from the divorce itself to its effects on parental well-being.

## Footnotes

1 Some studies have focused on parents but have not compared them with couples without children (e.g., Kamp Dush 2013).

2 This model is similar to the “role” model and the “chronic strain” model.

3 Because of our study focus, we considered only observations of respondents aged 60 or younger. Furthermore, we removed 92 divorcees from a selective high-income sample (Sample G, drawn in 2002).

4 We removed the observation directly preceding divorce because well-being in this year is often affected by the impending marital breakup.

5 Replication files to this article are available online (www.thomasleopold.eu).

6 SOEP uses the educational levels given by the CASMIN classification (Comparative Analysis of Social Mobility in Industrial Nations). The distinction between East and West Germans was based on a survey question about where a respondent lived in 1989 (i.e., before reunification).
